# Novel computational analysis of protein binding array data identifies direct targets of *Nkx2.2 *in the pancreas

**DOI:** 10.1186/1471-2105-12-62

**Published:** 2011-02-25

**Authors:** Jonathon T Hill, Keith R Anderson, Teresa L Mastracci, Klaus H Kaestner, Lori Sussel

**Affiliations:** 1 Department of Genetics and Development, Columbia University, New York, NY 10032, USA; 2 Department of Biochemistry and Molecular Genetics, University of Colorado at Denver and Health Sciences Center, Aurora, CO 80045, USA; 3 Department of Genetics and Institute for Diabetes, Obesity and Metabolism, University of Pennsylvania, Philadelphia, PA 19104, USA

## Abstract

**Background:**

The creation of a complete genome-wide map of transcription factor binding sites is essential for understanding gene regulatory networks *in vivo*. However, current prediction methods generally rely on statistical models that imperfectly model transcription factor binding. Generation of new prediction methods that are based on protein binding data, but do not rely on these models may improve prediction sensitivity and specificity.

**Results:**

We propose a method for predicting transcription factor binding sites in the genome by directly mapping data generated from protein binding microarrays (PBM) to the genome and calculating a moving average of several overlapping octamers. Using this unique algorithm, we predicted binding sites for the essential pancreatic islet transcription factor *Nkx2.2 *in the mouse genome and confirmed >90% of the tested sites by EMSA and ChIP. Scores generated from this method more accurately predicted relative binding affinity than PWM based methods. We have also identified an alternative core sequence recognized by the *Nkx2.2 *homeodomain. Furthermore, we have shown that this method correctly identified binding sites in the promoters of two critical pancreatic islet β-cell genes, *NeuroD1 *and *insulin2*, that were not predicted by traditional methods. Finally, we show evidence that the algorithm can also be applied to predict binding sites for the nuclear receptor *Hnf4α*.

**Conclusions:**

PBM-mapping is an accurate method for predicting Nkx2.2 binding sites and may be widely applicable for the creation of genome-wide maps of transcription factor binding sites.

## Background

The dynamic process of gene regulation is essential for embryonic development and cellular function. Gene regulation is primarily mediated by the combinatorial effects of transcription factors interacting with *cis*-regulatory elements such as promoters and enhancers. Therefore, accurate identification of transcription factor binding sites within the genome is necessary to understand a wide range of cellular processes from cell differentiation to homeostasis to cancer. However, identifying these sites within the genome remains a complex biological and computational question.

One of the challenges in predicting transcription factor binding sites is that identification of the strongest binding sequence, or consensus site, is not sufficient. Research analyzing genome wide transcription factor occupancy has shown that low affinity binding sites are also significantly occupied in both yeast and *Drosophila *[1,2]. Furthermore, transcription factors from the same family have been shown to bind identical high affinity sites, but distinct low affinity sites [3,4]. Therefore, identification of both high and low affinity sites will be essential to fully understand transcription factor specificity within the genome.

Current transcription factor motif algorithms generally rely on a statistical model, such as a position weight matrix (PWM), generated with information derived from homology between co-regulated promoters, conserved regions in orthologous genes, known binding sites, or *in vitro *binding assays [5]. However, these methods have a low level of specificity and sensitivity [6-9]. This problem is due to both experimental and theoretical errors. Experimental errors include alignment of a limited set of binding sites [10], resulting in a PWM that is information poor, and non-physiological conditions used in *in vitro *binding assays [11]. Theoretical errors stem from assumptions used as the basis of the PWM model. PWMs assume that contributions by individual bases within a binding site are independent and additive, and that the binding energy contribution is proportional to their frequency in the position weight matrix. However, all three of these assumptions have been called into question. Interdependencies have been demonstrated at least in a subset of transcription factors resulting in contributions to binding affinity that are both interdependent and non-additive [10,12,13]. Position weight matrix scores have also been experimentally shown to be a poor predictor of binding affinities in both eukaryotic and prokaryotic systems [7,14].

*Nkx2.2 *is a homeodomain transcription factor expressed in the ventral neural tube, intestine and pancreas [15]. A consensus sequence (T(t/c)AAGT(a/g)(c/g)TT) has been identified by SELEX and a corresponding PWM was generated and deposited in the TRANSFAC database [16]. However, experiences in our lab and others have shown that the predictive power of this PWM is low. More recently, Berger et al. [4] generated a PWM for *Nkx2.2 *using protein binding microarray (PBM) technology. PBMs use a mathematically constructed set of oligos to quantitatively measure protein-DNA binding for all possible octamers. This should, in theory, result in a PWM that is more information rich than those constructed by other methods.

Here, we show the results of an in-depth analysis of the PBM data for *Nkx2.2*. The original *Nkx2.2 *consensus sequence contains an invariable "AAGT" core. We have identified an alternative low-affinity core sequence with a wobble in the first position to contain "GAGT". We also mapped the PBM data directly to the genome to identify putative *Nkx2.2 *binding sites. Using this method, we identified 111 novel *Nkx2.2 *binding sites within the proximal promoters of genes differentially expressed in wildtype and *Nkx2.2 *null pancreata and confirmed approximately 90% of these sites by EMSA and/or ChIP analysis. Six of the sites confirmed by EMSA and ChIP contain the alternative "GAGT" core sequence. We also show that using a moving average of E-scores from the protein binding microarrays to predict relative binding affinity outperformed both the TRANSFAC PWM and the PBM-based PWM. Since the PBM-based PWM and the method described in this study rely on the same input data, these results show that the assumptions used to generate PWMs do not accurately describe *Nkx2.2 *binding. Therefore, creating genome-wide maps by directly using experimental data will greatly increase the specificity and sensitivity of transcription factor binding site (TFBS) predictions over statistical models. Furthermore, these experiments revealed a gene battery that includes a large number of genes required for insulin secretion functions in the β-cell that is controlled by *Nkx2.2*. We demonstrate that this same method can be adapted to other transcription factors. Based on these findings, we propose that PBM-mapping can be used to create composite TFBS maps across the entire genome. Such a map would greatly aid in the identification of *cis*-regulatory elements and the understanding of gene regulation.

## Results

### Identification of an alternative Nkx2.2 binding site core sequence

*Nkx2.2 *was previously shown to specifically bind a 10 base-pair sequence containing an invariable "AAGT" core sequence flanked by several less conserved bases [16]. To date, only two *in vivo *binding sites (in the *insulin2 *and *MafA *promoters) identified using this sequence have been successfully verified [17,18]. One possible explanation for the low predictive power of this consensus sequence is that it does not completely encompass the possible binding motifs for *Nkx2.2 *binding. More recently, Berger et al. [4] published a protein binding microarray (PBM) analyzing the binding affinity of the *Nkx2.2 *homeodomain. PBMs generate an enrichment score (E-score) with a range from -0.5 (low affinity) to 0.5 (high affinity) for every possible 8-base combination based on the relative intensity readouts from microarray data [19]. Therefore, it has the potential to be more information rich than other methods because it allows for complete coverage of possible binding sequences and provides quantitative binding results. We used this data as a basis to identify additional *Nkx2.2 *DNA binding motifs.

**Figure 1:**
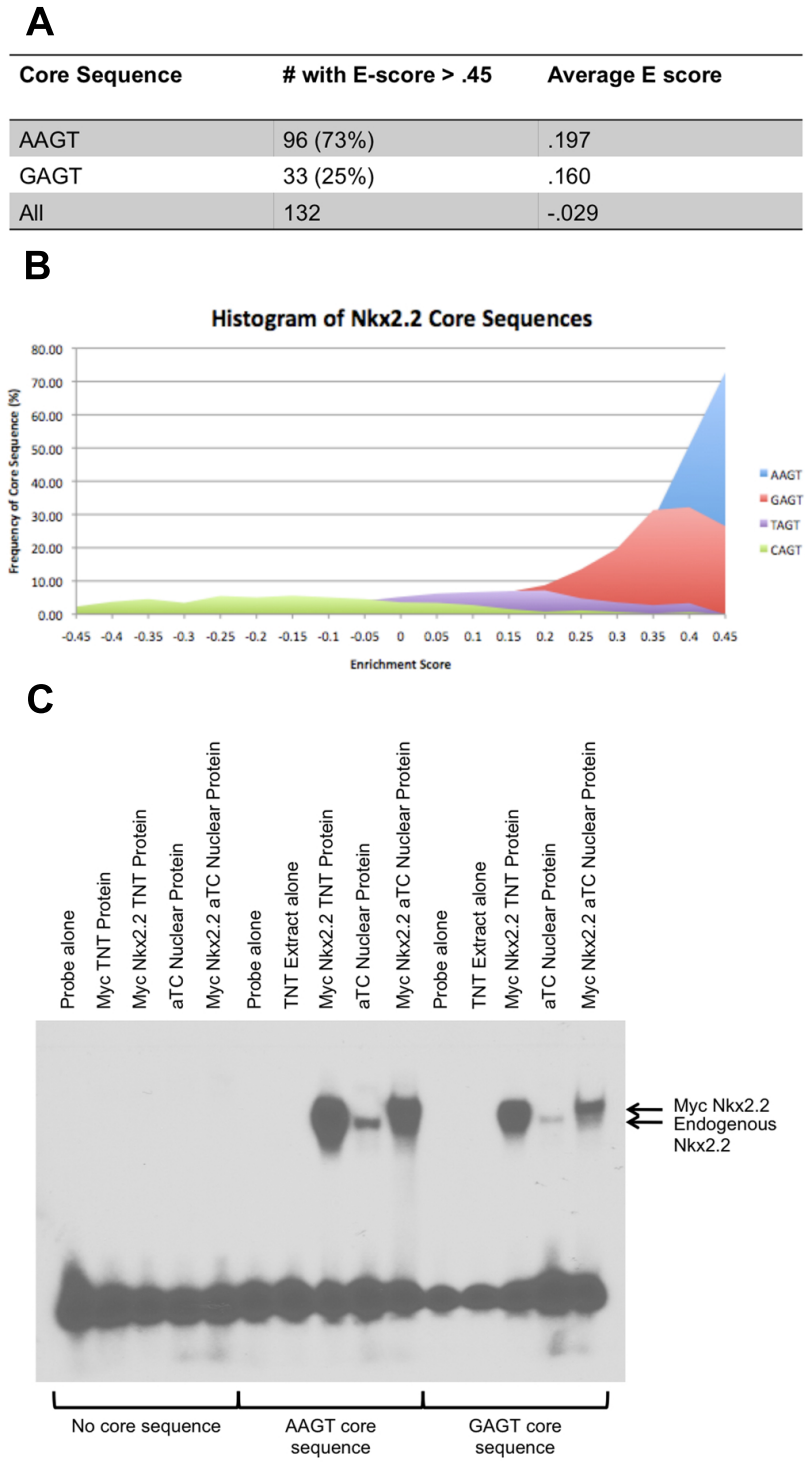
***Nkx2.2 *binds to the alternative core sequence "GAGT"**. (A) Table showing E-score distribution of octamers. E-scores were generated using protein binding microarray data. Octamers were divided into AAGT containing, GAGT containing and all octamers (left column). The number of octamers in each group with an E-score above 0.45 is shown in the middle column. Average E-score from all octamers in each group is shown in the right column. (B) Histogram plot of E-score distribution for AAGT, GAGT, TAGT and CAGT. Each point represents the percentage of total sites within a 0.10 bin that contain the given core sequence. (C) EMSA analysis of the canonical AAGT containing consensus probe (Sup. Table 3: "Nkx2.2 AAGT"), a GAGT core containing probe (Sup. Table 3: "Nkx2.2 GAGT"), and a probe with no core sequence (Sup. Table 3: "Nkx2.2 No Core"). Each probe was incubated with *in vitro *synthesized *Nkx2.2 *(Myc tagged-*Nkx2.2 *TNT Protein) or αTC1 nuclear extract with or without transfected Myc tagged-*Nkx2.2*.

We first selected and analyzed all *Nkx2.2*-bound octamers with an E-score greater than 0.45 (132 octamers, Figure 1A). Of these, 96 (73%) contained the previously published "AAGT" core sequence or its reverse complement. Of the remaining 36 octamers, 33 (25% of the total) had an alternative sequence "GAGT." Three octamers did not contain either core sequence. We next calculated the average E-score for octamers containing AAGT and octamers containing GAGT. The average of all possible octamers was used as a baseline control. AAGT and GAGT containing octamers had a mean E-score value of 0.197 and 0.160, respectively, which are significantly greater (P << 0.001) than the mean for all possible octamers (-0.029).

Several combinations of two core sequences are possible within a single octamer. In order to identify the effect of two adjacent cores on Nkx2.2 binding, we analyzed these "dual-core" octamers (Additional File 1). Dual-core octamers for Nkx2.2 binding can be divided into 4 groups based on which core sequence (AAGT or GAGT) is included and their relative orientation (inline or reverse compliment). Interestingly, all octamers containing two cores in the reverse compliment orientation had E-scores > 0.45 while octamers with inline cores had E-scores < 0.37, independent of which cores were contained in the octamer.

The two identified core sequence motifs differ only in the first position. In order to determine whether significant enrichment could be achieved with the other two possible first position bases, we plotted a histogram of the number of occurrences of each possible base in the first position for all E-scores (Figure 1B). We found that there is a significant enrichment of only the AAGT and GAGT core sequences.

To experimentally test the alternative GAGT binding site, we performed Electrophoretic Mobility Shift Assay (EMSA) experiments using the previously published *Nkx2.2 *consensus sequence as a baseline probe [16,20] (Figure 1C). We mutated this probe to contain the alternative GAGT core sequence (max E-score = 0.48593, PBM-mapping score = 0.43038) and compared binding to both the original consensus probe (max E-score = 0.49841, PBM-mapping score = 0.46186) and a probe with a deleted core sequence (max E-score = 0.11739, PBM-mapping score = 0.05029). The GAGT containing probe showed significant binding with *in vitro *translated *Nkx2.2 *(TNT *Nkx2.2*) or nuclear extract from pancreatic cell lines with or without exogenously expressed *Nkx2.2*, although binding was weaker than the AAGT containing probe. Taken together, these experiments show that GAGT represents an alternative core sequence for *Nkx2.2 *binding sites, although its relative binding affinity is lower than the canonical AAGT core sequence.

### Identification of Novel Endogenous Nkx2.2 Binding Sites Using Protein Binding Microarray Mapping

**Figure 2:**
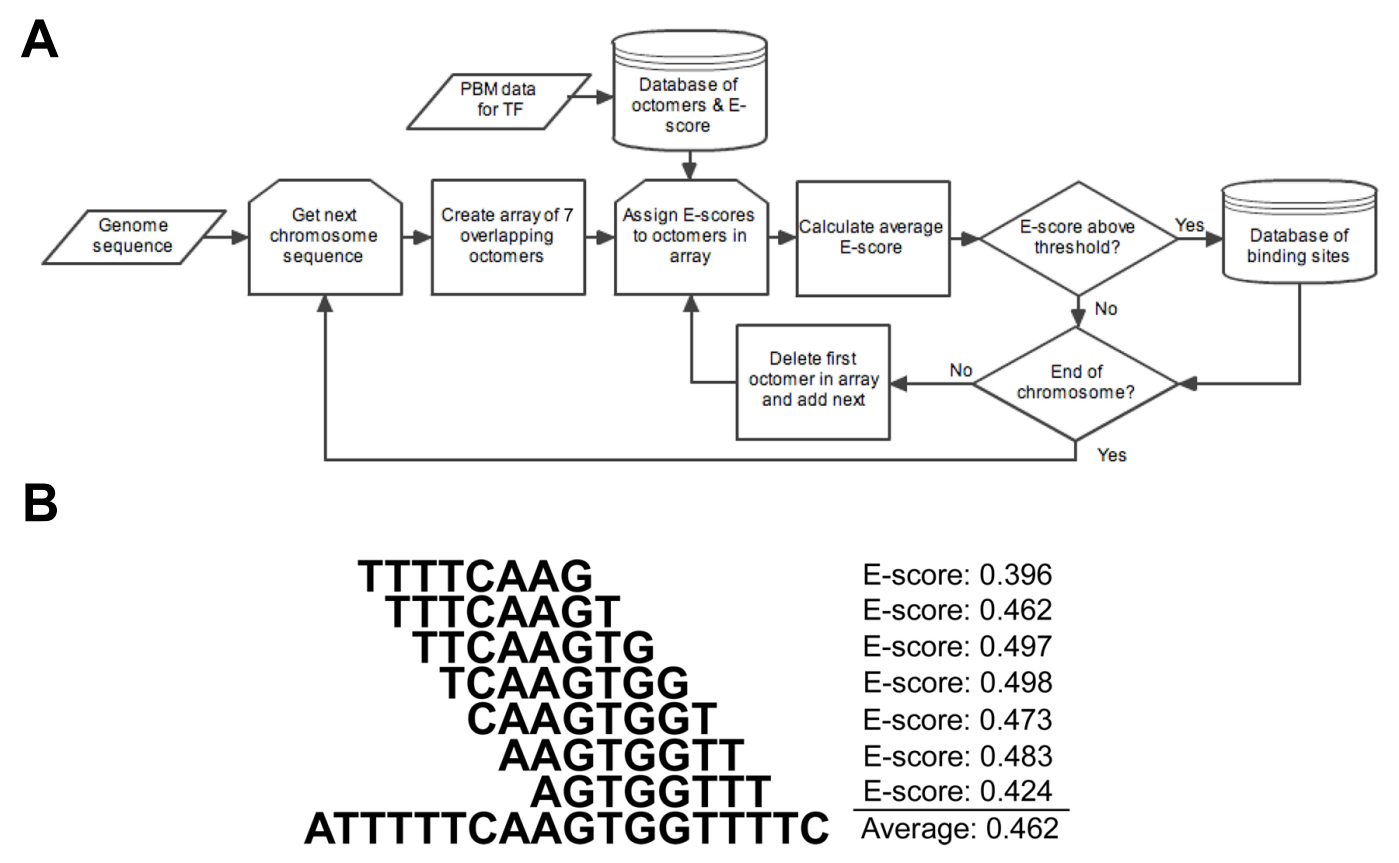
**Transcription factor binding site prediction using PBM mapping**. (A) Flow chart diagram outlining prediction method. (B) Diagram showing the calculation of the moving average of E-scores. A window containing 7 overlapping octamers was generated and the mean of the E-scores for each octamer was calculated. The sequence and score for the *Nkx2.2 *consensus sequence is shown.

Several methods to predict genomic transcription factor binding sites using PBM data have been developed. The most common methods rely on PWM creation by the "seed-and-wobble" algorithm or parametric methods [19,21]. However, these methods are limited by the inaccuracies of the PWM model. E-scores of single octamers have also been suggested to be correlated with transcription factor binding affinity, suggesting that they might be able to be directly used to predict binding sites [3]. However, the dataset of E-scores has been shown to be somewhat noisy [21]. In order to compensate for dataset noise, Grove et al. [22] used an average of 3 E-scores based on a perfectly conserved core sequence (the AvgES method). However, we show in this study that the core sequence may not be completely conserved. Therefore, we sought to develop a novel algorithm that utilizes a moving average of E-scores for overlapping octamers to predict endogenous *Nkx2.2 *binding sites without the bias for PWM-derived consensus core sequences and with more overlapping octamers than were used in the AvgES method (Figure 2). We refer to this method as PBM-mapping. Briefly, E-scores are mapped to each octamer in the genome and a moving average of several overlapping octamers is calculated. We experimentally determined that using a moving average of 7 octamers predicted binding affinity with the greatest accuracy (see below and Figure 2B). Sequences with a moving average higher than a determined threshold are then deposited into a database that can be queried to identify putative sites. We show by DNA binding assays that the threshold should be set to approximately 0.37 for *Nkx2.2*, although this threshold may vary for other transcription factors (see below).

We tested our algorithm by searching for Nkx2.2 binding sites throughout the genome. Complete analysis of the genome resulted in 3 × 10^6 ^predicted sites, which falls within range of the expected number of binding sites in the genome for a single transcription factor, based on statistical probability of the sequence occurring in the genome. In order to investigate sites that are most likely to be biologically relevant, we selected sites within regulatory regions from 2.5 kb upstream to 1 kb downstream of the transcription start site of 35 genes with expression levels significantly (P ≤ 0.05) changed in *Nkx2.2 *null embryos [23-25]. A total 111 putative binding sites were identified in 31 differentially expressed genes including 7 of the 8 genes with increased expression in the *Nkx2.2 *null pancreas, and 24 of the 27 genes with decreased expression in the *Nkx2.2 *null pancreas. Furthermore, genes with differential expression in the Nkx2.2 null embryo were more likely to have predicted sites within 500 bp upstream of the transcriptional start site (Additional File 2, P = .02).

**Figure 3:**
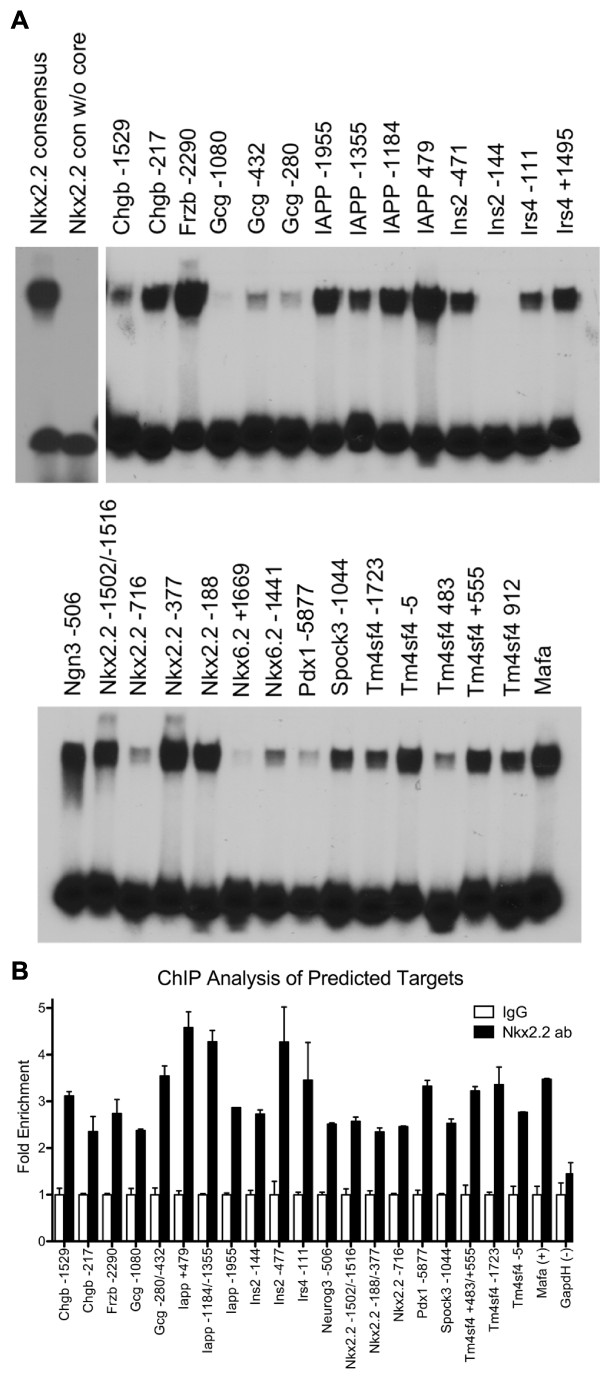
**Confirmation of PBM-mapping predicted sites by EMSA and ChIP Analysis**. (A) EMSA analysis of selected predicted sites. Probes spanning approximately 50-60 bp surrounding the predicted site were incubated with *in vitro *synthesized *Nkx2.2*. The *Nkx2.2 *consensus probe and the consensus probe with the core sequence deleted were used as positive and negative controls, respectively. (B) Confirmation of *in vivo *promoter occupancy at predicted sites by ChIP. βTC6 cells were used for chromatin input and the *Nkx2.2 *mouse monoclonal antibody was used for precipitations. Enrichment is shown as fold change over IgG. All predicted sites were significantly increased over the IgG control (P < 0.05). The housekeeping gene GapdH was used as a negative control and was not significantly enriched.

We chose to test the binding of 24 putative sites that were randomly selected from the promoters of differentially expressed genes using EMSA analysis (Figure 3A). We also included 3 predicted sites that were located outside of the designated promoter region because they fell in genes of particular interest, including a site in the recently described Region IV enhancer of the *Pdx1 *promoter [26] and additional sites in the promoter regions of *Irs4 *(Irs4 +1495) and *Nkx6.2 *(Nkx6.2 +1669). In addition, we tested a previously published *Nkx2.2 *binding site in the *insulin2 *promoter (Ins2 -144) that is the only previously published *Nkx2.2 *site not predicted by PBM-mapping [17]. The published *Nkx2.2 *site in the MafA promoter (Mafa -7762, PBM-mapping score = 0.417) was used as a positive control [18]. Of the 28 sites tested by EMSA, only the Ins2 -144, the Nkx6.2 +1669, and the glucagon -1080 sites did not show detectable binding. Consistently, the Gcg -1080 and Nkx6.2 +1669 sites had averages E-score of 0.347 and 0.364, respectively, and represented the lowest scores of any predicted site tested. The Ins2 -144 site was below our original threshold with an average E-score of 0.233.

Although EMSA analysis shows the sequence specificity of transcription factor binding, it does not take into account cell-specific chromatin states or cooperative factors that may affect binding *in vivo*. In order to confirm *Nkx2.2 *occupancy of these sites in β-cells, we performed chromatin immunoprecipitation (ChIP) using the βTC6 cell line (Figure 3B). We did not include the Nkx6.2 -1441, Nkx6.2 +1669, Irs4 +1495 and Tm4sf4 +912 sites in this analysis because they fell in low complexity regions, which hindered the design of appropriate primers. Precipitation with the *Nkx2.2 *monoclonal antibody resulted in significant enrichment of all predicted promoters that were tested (Figure 3B). The promoter for the housekeeping gene *GapdH *was used as a negative control. This data shows that promoters containing predicted sites are occupied *in vivo*. However, ChIP results cannot distinguish between multiple sites in of the *Gcg, Ins2, Iapp, Nkx2.2*, and *Tm4sf4 *promoters due to the close proximity of the sites.

### PBM-mapping accurately predicts relative Nkx2.2 binding affinity in vitro

**Figure 4:**
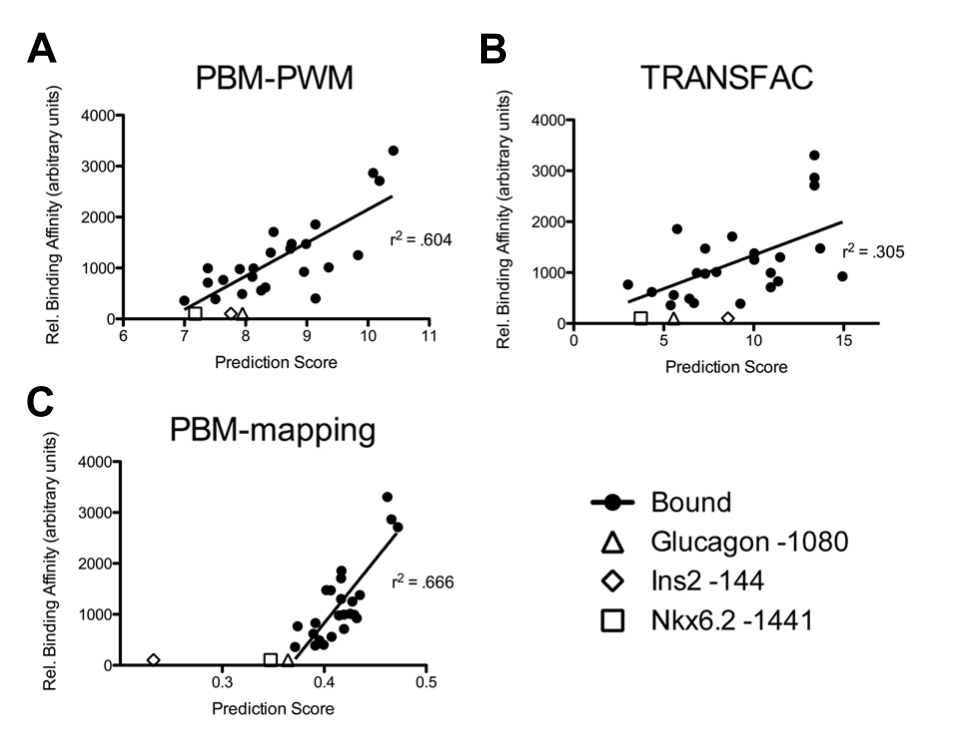
**Linear regression of various prediction methods and relative binding affinity**. In each panel, the highest score obtained from the EMSA probe was compared to relative binding affinity (fraction bound) calculated from the EMSA in Figure 2. Probes with more than one predicted site (Spk3 -1044 and Nkx2.2 -1503) were excluded. Scores from probes that were not bound in the EMSA (Gcg -1080, Nkx6.2 +1669, and Ins2 -144) were plotted along the X-axis and not used for r-squared calculation. Scores used were (A) average e-score from 7 overlapping octamers from PBM-mapping, (B) log-odds from TRANSFAC-PWM, and (C) Seed and Wobble matrix score from PBM-PWM.

Transcription factor binding *in vivo *is not a binary event but a continuum of site occupancy proportional to the binding affinity (K_a_) of the transcription factor and its binding site. Therefore, the ideal TFBS prediction algorithm would generate a score that is highly correlated with transcription factor binding affinity. It has been proposed that the E-score from PBM experiments is indicative of relative binding affinity and preliminary experiments have shown correlation between individual octamer E-scores and binding affinity [3,21]. Therefore, in order to test whether single octamer and average E-scores are correlated with relative *Nkx2.2 *binding affinity, we quantified the fraction bound for each site in the EMSA analysis (normalized to the probe with the largest bound fraction) and graphed it against single E-scores for the highest octamer and averages of 3, 5, 6, 7 or 8 oligos (Additional File 3). The fractional occupancy of a transcription factor bound to a DNA binding site is indicative of the relative binding affinities of the ligands [27]. The average of 7 overlapping scores showed the highest correlation with relative binding affinity (r-squared = 0.666) and outperformed both the TRANSFAC PWM score (r-squared = 0.305) and the PBM seed and wobble matrix score (r-squared = 0.604) (Figure 4). In order to confirm the correlation between the PBM-mapping score and biochemically-derived binding affinity values, we analyzed 22 binding-sites with K_d _values that were determined for the *Nkx2.2 *drosophila homolog, *vnd *[28]. The homeodomains of the fly and mouse proteins contain 95% amino acid identity and greater than 98% similarity, therefore the K_d _values for *Nkx2.2 *and *vnd *should also be very similar. Regression analysis of PBM-mapping scores against the K_d _values for 22 *vnd *sites showed strong correlation (r^2 ^= 0.83, Additional File 4). Taken together, these experiments show that PBM-mapping represents a highly accurate prediction method to find genome wide binding sites.

### AAGT and GAGT core sequences contribute to Nkx2.2 binding in the NeuroD1 promoter

**Figure 5:**
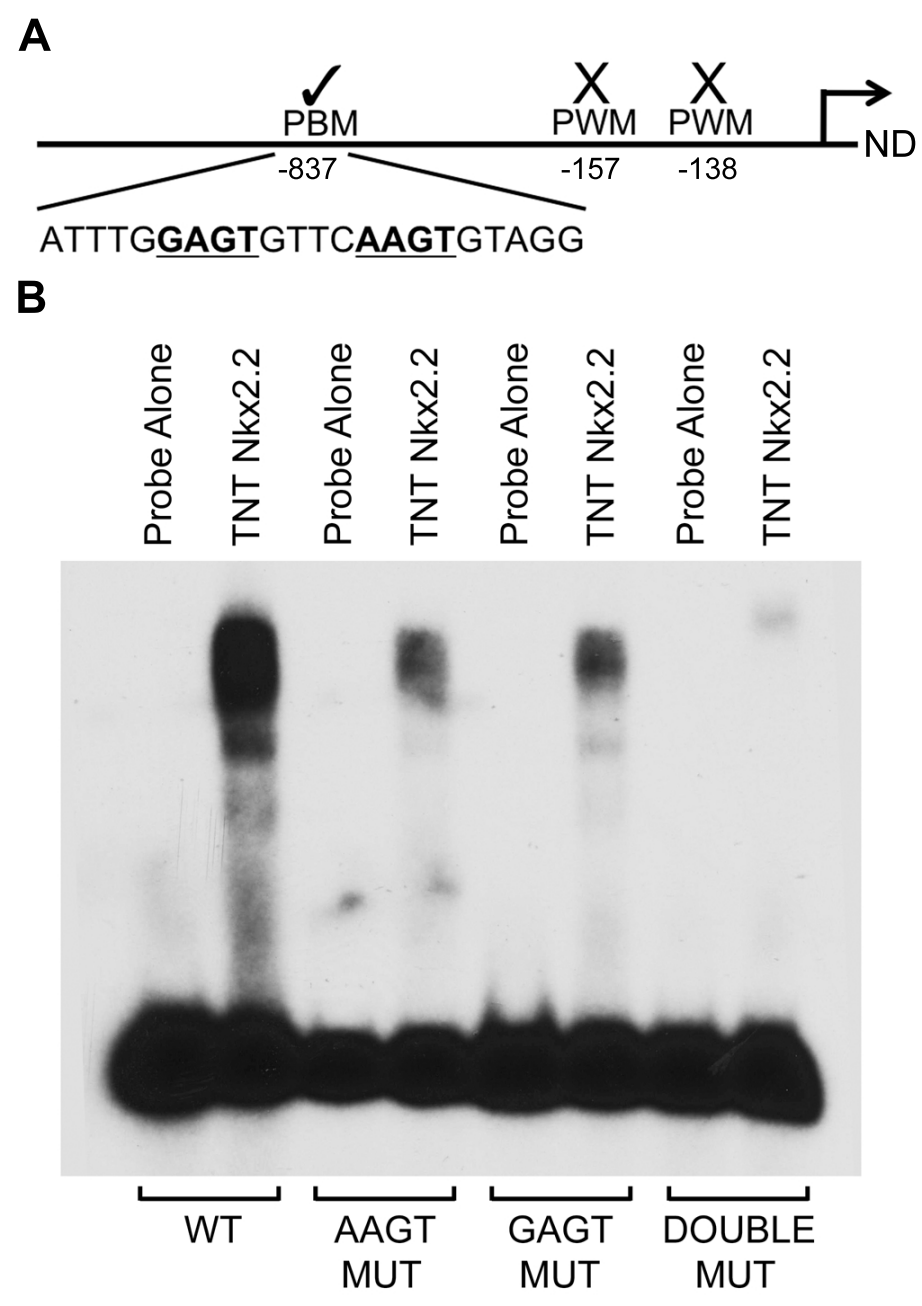
***Nkx2.2 *binding to the *NeuroD1 *promoter through GAGT and AAGT sites**. (A) Schematic representation of the *NeuroD1 *promoter. The TRANSFAC PWM predicted two sites that were not bound *in vitro *or *in vivo *[20]. PBM-mapping predicted a novel site upstream of the other two sites that contains both a GAGT core and an AAGT core separated by 4 bp. (B) EMSA analysis showing binding through both core sites. Wildtype, AAGT mutant, GAGT mutant and double mutant probes were incubated with *in vitro *translated *Nkx2.2*.

Although there is overlap between PWM based predictions and PBM mapping, the predictions are significantly different for sites within the *NeuroD1 *gene, which have previously been shown to be directly regulated by *Nkx2.2 *[20]. In the *NeuroD1 *promoter, previous analysis for *Nkx2.2 *binding predicted two sites that could not be confirmed by EMSA analysis [20](Figure 5A). PBM-mapping did not predict either of these sites, but predicted a novel site at -837 that was bound in EMSA experiments (Figure 5B, see also [20]). The PBM-mapping predicted *Nkx2.2 *site within the *NeuroD1 *promoter is unique because it is predicted to consist of two adjacent *Nkx2.2 *binding sites (Figure 5A). One binding site contains the previously published "AAGT" core sequence while the other has the novel "GAGT" core sequence identified in this study. Mutation of each individual core sequence showed a reduction in binding while mutation of both cores simultaneously was necessary to completely ablate *Nkx2.2 *binding (Figure 5B), suggesting that both cores contribute to *Nkx2.2 *binding. Interestingly, we did not detect a slower migrating protein complex forming on the double site, suggesting that dimer formation is prevented, possibly by steric hindrance. This may represent a unique mechanism to increase transcription factor occupancy on the promoter.

### Identification of a novel Nkx2.2 binding site in the insulin promoter

**Figure 6:**
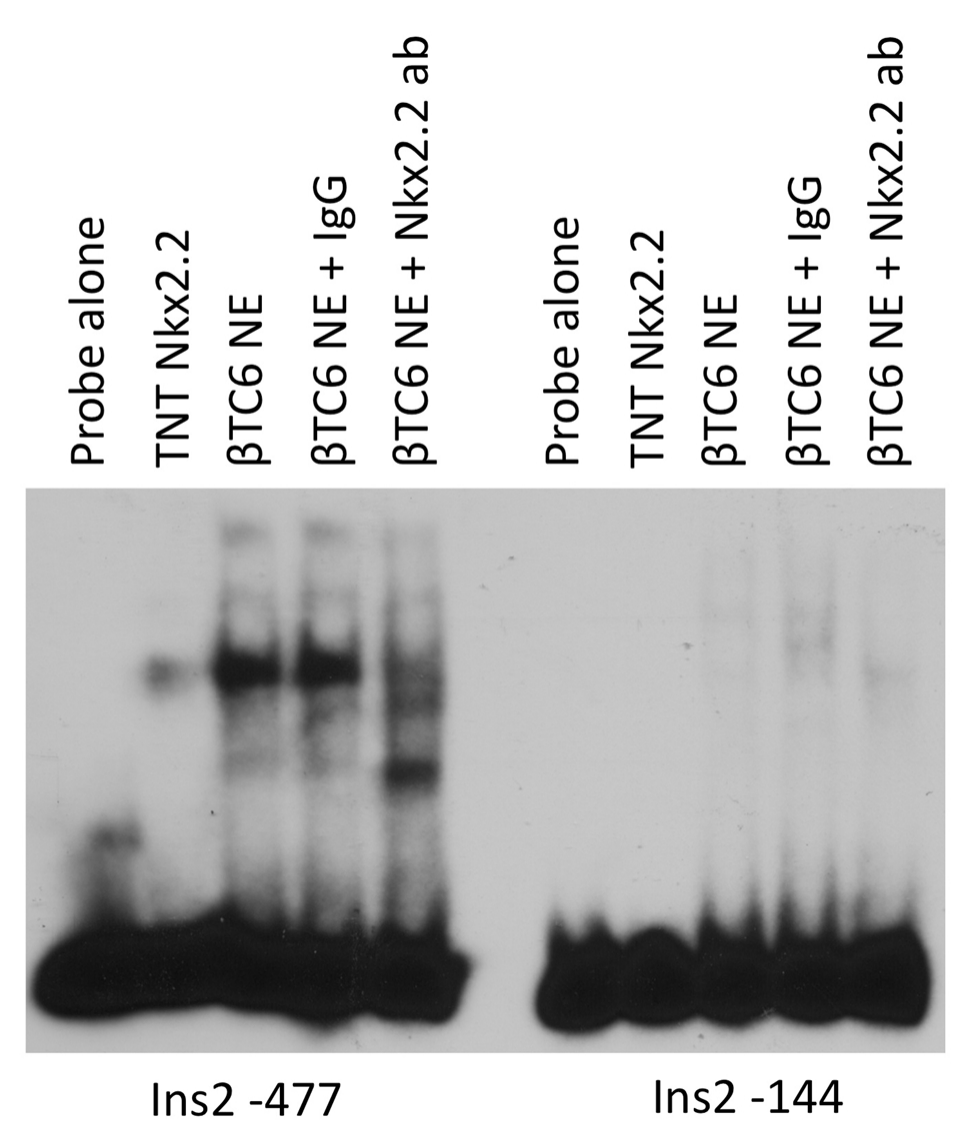
***Nkx2.2 *binds the Ins2 promoter through a novel site**. EMSA analysis of putative *Nkx2.2 *binding sites in the Ins2 promoter. Probes were incubated with *in vitro *translated *Nkx2.2 *or bTC6 nuclear extract. Supershifts were done using the monoclonal *Nkx2.2 *antibody.

An *Nkx2.2 *binding site in the insulin promoter (Ins2 -144) was previously identified [17]. This site is predicted by the TRANSFAC PWM and the PBM seed and wobble matrix, but is not predicted by PBM-mapping (Figure 4). We were unable to confirm *Nkx2.2 *binding to the previously published site by *in vitro *generated *Nkx2.2 *(Ins2 -144, Figure 3A). However, PBM mapping predicted a site 328 bases upstream of the previously published site that was confirmed by EMSA (Ins2 -477, Figure 3A). ChIP analysis showed *Nkx2.2 *occupancy with primers for both the published and the newly predicted site, although occupancy appeared to be stronger on the PBM-mapping predicted site (Figure 3B). Although the ChIP results are unable to completely distinguish between occupancy of both sites because of their close proximity relative to the DNA fragmentation size, it is also possible that *Nkx2.2 *binds the Ins2 -144 site through cooperative binding with cofactors that would not have been detected by EMSA analysis using *in vitro *translated *Nkx2.2*. Therefore, we performed additional EMSA analysis using βTC6 nuclear extract (Figure 6, Additional File 5). A strong shift was observed when βTC6 nuclear extract was incubated with the Ins2 -477 probe and this shift was inhibited by addition of *Nkx2.2 *antibody (Figure 6). A weaker *Nkx2.2 *containing complex could also be detected bound to the Ins2 -144 site (Figure 6 and Additional File 5). Therefore, it appears that *Nkx2.2 *may be stabilized on the Ins2 -144 site by interacting factors, although this interaction appears to be much weaker than the -477 site identified by PBM-mapping.

### PBM-mapping accurately predicts Hnf4α binding sites

To test the wider applicability of PBM-mapping for the general prediction of transcription factor direct targets, we applied a moving average of E-scores from 6 overlapping octamers to the nuclear receptor family member *Hnf4α*. *Hnf4α *has been shown to bind DNA as a homodimer to a site containing two direct repeats of the consensus binding site (consensus site: AGGTCA) [29]. Therefore, *Hnf4α *represents a transcription factor that is structurally and functionally distinct from *Nkx2.2*. We applied PBM-mapping to 30 putative *Hnf4α *binding sites (18 positive and 12 negative) that were previously tested by EMSA analysis [30]. At a threshold value of 0.26, PBM-mapping predicted 16 of the 18 (89%) confirmed binding sites and correctly did not predict any of the negative sites (Additional File 6). Adding or subtracting the number of overlapping octamers did not improve prediction scores for the analyzed data set (data not shown). This data suggests that PBM-mapping may be directly applicable to a wide range of transcription factors.

## Discussion

The identification of transcription factor binding sites is an important biological question. To date, the majority of methods to detect these sites have focused on creating statistical models, such as position weight matrices, of transcription factor specificities. However, these models are limited due to the fact that they must make generalized assumptions about transcription factor binding properties that are not completely understood. Other recent technologies have been developed such as ChIP-Seq to look at genomic transcription factor occupancy [31]. However, these technologies do not identify the precise binding sites, are technically difficult, and limited by the lack of high quality antibodies for many transcription factors. Therefore, bioinformatic prediction of transcription factor binding sites remains a powerful and useful tool for understanding transcriptional regulation. In this paper, we propose a modified technique for creating genome-wide TFBS site maps using direct mapping of protein binding microarray data (PBM-mapping). This method is technically simpler than ChIP-seq methods and does not rely on the assumptions used in statistical models.

We have shown that PBM-mapping more accurately predicts relative binding affinity than previously reported TRANSFAC or PBM based PWMs. PWM inaccuracies have been attributed to both experimental and theoretical errors [32]. Our studies support both of these limitations. The TRANSFAC PWM was developed from the alignment of 23 sequences enriched using SELEX experiments. The PBM-PWM was based on microarray experiments, which provide data for all possible octamers. In our experiments, the PBM-PWM was more highly correlated with observed relative binding affinity than the TRANSFAC PWM, most likely due to the increased information content in the PBM-PWM. However, PBM-mapping scores correlated to a greater extent with relative binding affinity than either PWM method, even though it was generated from the same data used for the PBM-PWM. Therefore, the PWM statistical model does not accurately model *Nkx2.2 *binding.

Numerous methods for generating PWMs exist and there are several statistical corrections that can be applied to the PWM model, however accurately testing and comparing all of these corrections is technically difficult and therefore were outside the scope of this study [33]. Predictions based on the combined results of more than one PWM could also be attempted. However, these predictions would still be susceptible to the limitations of the PWM model to account for the influence of neighboring nucleotides and flanking regions.

A method for using the average E-score of 3 overlapping octamers surrounding a previously identified core sequence (AvgES) has been utilized for predicting binding sites in *C. elegans *[22]. However, this method was not fully tested for accuracy and is biased by the assumption of a completely conserved 4-basepair long core sequence. Our results show that using an average E-score from an increased number of overlapping octamers improved the accuracy of transcription factor binding site prediction. Interestingly, an average E-score of 7 overlapping octamers resulted in the highest correlation with relative binding affinity. This represents the greatest number of overlapping octamers possible with at least one base pair common between all oligos. *Nkx2.2 *binding has been reported to be influenced by the flanking DNA sequence [16,34]. Therefore, the increase in accuracy is most likely due to accounting for the flanking regions; however, it may also be due to reductions in the inherent noise present from microarray quantification [21]. It remains possible that bases outside of the 7 octamer window can affect binding affinity, although using a window larger than 7 overlapping octamers resulted in decreased accuracy. This may be due to limitations of using 8 bp long motifs for generation of PBM data. It remains to be seen if increasing the length of DNA-binding sequences used in the microarray based experiments to generate E-scores would further increase the accuracy of binding affinity prediction.

We did not observe a large difference between the flanking regions of "AAGT" and "GAGT" containing sequences. Furthermore, both core sequences share common flanking regions in the octamers with the highest E-score (TC**AAGT**GG and TC**GAGT**GG). However, the magnitude of the effect that base substitutions in the flanking regions have of the overall score of the site differed between "AAGT" and "GAGT" containing sites. For example, conversion of "G" to "C" in the last position of the above mentioned octamers reduces the single octamer E-score of the "AAGT" site by 0.0038 and the "GAGT" site by 0.011, confirming that the bases in these positions are not independently additive, but are dependent on other bases in the binding site, even when these bases are not immediately adjacent.

We tested PBM-mapping using the homeodomain transcription factor *Nkx2.2*. *Nkx2.2 *has been shown to act as both an activator and a repressor during pancreatic islet formation [23,35]. β-cells are completely absent in the *Nkx2.2 *null embryo [15]. There was also a corresponding decrease in many, but not all, β-cell markers [25]. However, it was unclear which of these genes were down-regulated due to direct transcriptional control by *Nkx2.2 *or because the β-cell population was absent. We used PBM-mapping to predict novel *Nkx2.2 *binding sites within bound promoters of genes differentially expressed between wildtype and *Nkx2.2 *null embryos. Interestingly, a large majority of differentially expressed genes had predicted sites, suggesting that between e12.5 and e13.5 the changes seen in the *Nkx2.2 *null pancreas are largely due to direct regulation by *Nkx2.2 *and not a cascade of downstream transcription factors. We were also able to predict binding sites in several β-cell specific genes, including a battery of genes encoding proteins present in secretory vesicles, such as insulin, IAPP and ChgB that appear to be coordinately activated by *Nkx2.2*.

Several studies have shown that relatively weak or secondary binding sites are biologically important [1,2]. These sites may create genomic binding profiles dependent on protein concentrations or they may differentially bind closely related transcription factors that share a common primary binding motif [3]. Careful analysis of the PBM data for *Nkx2.2 *revealed a previously unidentified alternative "GAGT" binding site motif for *Nkx2.2*. GAGT containing sites were also represented in our predicted sites (Gcg -432, Iapp -1355, *Nkx2.2 *-716, and Tm4sf4 -1723)--confirming the ability of the algorithm to predict secondary sites. This is the first secondary motif that has been identified for an Nkx2 family member, although a unique secondary motif has been identified for *Nkx3.1 *(GTAC)[3]. PBM-mapping does not predict that Nkx2.2 binds the GTAC core sequence; however this is not surprising since the homeodomain sequences between Nkx2 and Nkx3 family members are not well conserved and the two protein families are known to preferentially bind different core sequences [36]. Further research is necessary to determine whether the GAGT motif identified in this study is unique to *Nkx2.2 *or is shared among several Nkx2 genes.

The importance of weak or secondary binding sites highlights the importance of finding a suitable threshold for determining positive prediction results. In our study, we set the threshold for putative *Nkx2.2 *binding sites at 0.37 based on the results from our EMSA analysis. However, this may not accurately reflect *in vivo *binding. For example, the Gcg -1080 site, which had an average E-score just below our threshold, did not show binding in EMSA analysis (Figure 3A), but appeared to be occupied in ChIP (Figure 3B). Therefore, it is possible that our threshold is overly stringent or that *Nkx2.2 *binding to weak sites is dependent on the presence of cofactors. The appropriate threshold may also vary between cell types due to different expression levels of the transcription factor. Furthermore, our results show that the appropriate threshold for different transcription factors will differ.

## Conclusions

Although our studies to test PBM-mapping mainly focused on *Nkx2.2*, we believe that it could be widely applied to other transcription factor binding sites. Homeodomain proteins generally bind to a motif with a strong 4-5 base pair core with less conserved flanking sequences [37]; therefore, the PBM-mapping algorithm should be directly applicable to most homeodomain containing proteins. We also show that PBM-mapping can be applied to the nuclear receptor protein *Hnf4α*, but with a different threshold score and number of overlapping octamers than *Nkx2.2*. Not surprisingly, this suggests that the different DNA-binding site profiles associated with each class of protein will necessitate modifying the number of overlapping octamers used in each analysis. This will especially apply to transcription factor classes that contain shorter or longer binding sites. For example, several zinc finger proteins are thought to have binding sites that contains a 3-base pair core, while some bHLH factors have a 6 bp core sequence [38]. A shorter core sequence may rely more heavily on flanking sequences for binding specificity, which would require increased octamer coverage, whereas a longer core may be more self-contained and require fewer octamers for predictive coverage. Threshold values may also need to be adjusted for each protein analyzed. Therefore, adapting this method to other transcription factor families may require a training set of known binding sites to determine an appropriate threshold for each transcription factor. If previously identified binding sites are not available, a training set can be generated from a subset of PBM-mapping predicted sites that span a wide range of PBM-mapping scores, as was done in this study. Although small adaptations may be necessary, the application of this method to other transcription factors merits further investigation.

## Methods

### Alternative Core Sequence Identification

All octamers with an E-score greater than 0.45 were selected from the protein binding microarray for *Nkx2.2 *[4]. Octamers containing "AAGT" or its reverse compliment "ACTT" were removed and the remaining octamers analyzed for common motifs.

### *Nkx2.2 *and *Hnf4α *Binding Site Prediction

An algorithm to predict *Nkx2.2 *binding sites was developed and implemented in the Perl programming language as follows: Chromosome sequence data were retrieved from the UCSC genome browser website (http://genome.ucsc.edu). A moving average of the E-scores from the *Nkx2.2 *or *Hnf4α *protein binding microarrays was calculated for each subset of 7 overlapping octamers across the entire genome. Genomic locations of putative sites were then saved to a database. For *Nkx2.2*, results with an average E-score greater than 0.34 were originally considered positive, but subsequently modified to 0.37. For *Hnf4α*, a threshold value of 0.24 was used.

### Electrophoretic Mobility Shift Assay (EMSA)

*In vitro *synthesized *Nkx2.2 *protein was made using the TNT Coupled Reticulolysate System (Promega). Nuclear extract was prepared from βTC6 cells using the Nuclear Extract kit (Active Motif). Probes were designed for each of the predicted binding sites analyzed (Additional File 7). Binding reactions were performed as described by [20]. Shifted bands were quantified using the integrated mean of a fixed window for each of the shifts using Photoshop Extended CS3 (Adobe). Fraction bound was calculated in order to estimate the relative binding affinities using densitometry analysis of bound DNA compared to total DNA as measured by EMSA analysis.

### Regression Analysis

Regression analysis was performed using Prism 5 (Graphpad). Linear regression was used for PBM-mapping score/fraction bound comparisons. Fraction bound is related to K_d _by the equation K_d _= [ligand](1-Fraction Bound)/Fraction Bound. Therefore, the PBM-score should be correlated with the K_d _by the equation K_d _= z(1-(ax + b))/(ax + b). Non-linear regression of PBM-score predictions of Kd were performed using this equation as the model to create a best-fit trendline.

### Chromatin Immunoprecipitation (ChIP)

ChIP experiments were performed using the ChIP IT Express kit (Active Motif). βTC6 cells were grown in DMEM supplemented with 15% FBS. Approximately 1.5 × 10^7 ^cells were crosslinked in 1% paraformaldehyde for 10 min at room temperature. Chromatin was then extracted and sheared by sonication using a Diagnode BioRuptor (8min - 30sec ON/OFF) resulting in chromatin fragments from 200-800 bp long. The sheared chromatin was divided into 6 reactions and run independently. Immunoprecipitations were performed with 3 μg mouse anti-*Nkx2.2 *monoclonal antibody (DSHB). Normal mouse IgG (Millipore) was used as a negative control. Occupancy of the predicted sites was tested by Sybr-Green qPCR of two independent immunoprecipitations (primers are listed in Additional File 8).

## Authors' contributions

JTH conceived of and generated all code for the PBM-mapping algorithm, performed EMSA and ChIP analyses, performed statistical analysis on score correlations, and wrote the manuscript. KRA analyzed and performed EMSA and ChIP analyses for the NeuroD promoter. TLM collected RNA for a new microarray analysis (ArrayExpress E-MTAB-356). KHK participated in the microarray study and corresponding data analysis. LS participated in the study design and coordination and writing of the manuscript. All of the authors have read and approved the final manuscript.

## Supplementary Material

Additional file 1 **Analysis of Dual Core Sites**. Octamers containing two adjacent 4-bp core sequences were divided into groups based on content (2 identical cores or a mixture of AAGT and GAGT cores) and relative orientation (inline or reverse complement). Corresponding E-scores form PBM analysis are also shown. Octamers with two reverse complement cores consistently have higher E-scores than inline octamers regardless of content. Click here for file

Additional file 2 **Enrichment of sites in proximal promoter regions of genes differentially expressed in the *Nkx2.2 *null embryo**. Putative *Nkx2.2 *binding sites were predicted (PBM-mapping score > 0.40) in promoter regions of 100 randomly chosen genes from genes with no differential expression between the *Nkx2.2 *null and wildtype embryos and compared to the 35 differentially expressed genes. The distance from the transcriptional start site of the closest predicted site was then calculated. Differentially expressed genes were more likely to have sites within 500 bp of the transcriptional start site (P = 0.02). No statistically significant difference was seen in the other regions. Click here for file

Additional file 3 **Optimization of moving average of E-score values**. A moving average of E-scores containing 1, 3, 5, 6, 7, or 8 overlapping octomers was calculated and compared to relative binding affinity of each site. R-squared values are plotted next to each plot. The three sites that did not bind in our EMSA analysis are plotted along the x-axis to show their predicted scores compared to bound sites, but were not used to calculate r-squared values. Click here for file

Additional file 4 **PBM-mapping scores are highly correlated with K_d _values for the *Nkx2.2 *drosophila homolog *vnd***. Previously published K_d _values for 22 *vnd *binding sites were plotted against their respective PBM-mapping scores. Non-linear regression was performed using a previously derived equation for the expected relationship between PBM-mapping scores and K_d _values (see Methods). Click here for file

Additional file 5 **An *Nkx2.2 *containing complex forms on the Ins2 -144 site**. Longer exposure (48 hrs) of the EMSA analysis of putative Nkx2.2 binding sites in the Ins2 promoter shown in Figure 6. Probes were incubated with *in vitro *translated Nkx2.2 or βTC6 nuclear extract. Supershifts were done using the monoclonal Nkx2.2 antibody. Click here for file

Additional file 6 **Confirmation of previously tested Hnf4α sites**. PBM-mapping scores were generated for 18 positive and 12 negative Hnf4α sites that were previously published (28). At a threshold of 0.26, 16 of the 18 confirmed sites were predicted while all of the negative sites were not predicted. The two sites that were not predicted, but were bound in EMSA analysis, are highlighted in Bold. Click here for file

Additional file 7 **List of probes used in EMSA analysis**. Forward and reverse single stranded oligos that were annealed to form double stranded DNA probes with 5' overhangs. Probes were then labeled by Klenow extension to insert a ^32^P containing dCTP (see Methods). Click here for file

Additional file 8 **List of primers used for qPCR reactions**. PCR primers were designed to amplify an approximately 200 bp region flanking predicted *Nkx2.2 *binding sites (see Methods). Click here for file

## References

[B1] Segal E Raveh-Sadka T Schroeder M Unnerstall U Gaul U Predicting expression patterns from regulatory sequence in Drosophila segmentation Nature 2008 451 7178 535 540 10.1038/nature06496 18172436

[B2] Tanay A Extensive low-affinity transcriptional interactions in the yeast genome Genome Res 2006 16 8 962 972 10.1101/gr.5113606 PMC1524868 16809671

[B3] Badis G Berger MF Philippakis AA Talukder S Gehrke AR Jaeger SA Chan ET Metzler G Vedenko A Chen X Diversity and complexity in DNA recognition by transcription factors Science 2009 324 5935 1720 1723 10.1126/science.1162327 PMC2905877 19443739

[B4] Berger MF Badis G Gehrke AR Talukder S Philippakis AA Pena-Castillo L Alleyne TM Mnaimneh S Botvinnik OB Chan ET Variation in homeodomain DNA binding revealed by high-resolution analysis of sequence preferences Cell 2008 133 7 1266 1276 10.1016/j.cell.2008.05.024 PMC2531161 18585359

[B5] Elnitski L Jin VX Farnham PJ Jones SJ Locating mammalian transcription factor binding sites: a survey of computational and experimental techniques Genome Res 2006 16 12 1455 1464 10.1101/gr.4140006 17053094

[B6] Frech K Quandt K Werner T Finding protein-binding sites in DNA sequences: the next generation Trends Biochem Sci 1997 22 3 103 104 10.1016/s0968-0004(97)01006-2 9066261

[B7] Robison K McGuire AM Church GM A comprehensive library of DNA-binding site matrices for 55 proteins applied to the complete Escherichia coli K-12 genome J Mol Biol 1998 284 2 241 254 10.1006/jmbi.1998.2160 9813115

[B8] Stormo GD DNA binding sites: representation and discovery Bioinformatics 2000 16 1 16 23 10.1093/bioinformatics/16.1.16 10812473

[B9] Tompa M Li N Bailey TL Church GM De Moor B Eskin E Favorov AV Frith MC Fu Y Kent WJ Assessing computational tools for the discovery of transcription factor binding sites Nat Biotechnol 2005 23 1 137 144 10.1038/nbt1053 15637633

[B10] O'Flanagan RA Paillard G Lavery R Sengupta AM Non-additivity in protein-DNA binding Bioinformatics 2005 21 10 2254 2263 10.1093/bioinformatics/bti361 15746285

[B11] Djordjevic M Sengupta AM Shraiman BI A biophysical approach to transcription factor binding site discovery Genome Res 2003 13 11 2381 2390 10.1101/gr.1271603 PMC403756 14597652

[B12] Bulyk ML Johnson PL Church GM Nucleotides of transcription factor binding sites exert interdependent effects on the binding affinities of transcription factors Nucleic Acids Res 2002 30 5 1255 1261 10.1093/nar/30.5.1255 PMC101241 11861919

[B13] Man TK Stormo GD Non-independence of Mnt repressor-operator interaction determined by a new quantitative multiple fluorescence relative affinity (QuMFRA) assay Nucleic Acids Res 2001 29 12 2471 2478 10.1093/nar/29.12.2471 PMC55749 11410653

[B14] Liu J Stormo GD Combining SELEX with quantitative assays to rapidly obtain accurate models of protein-DNA interactions Nucleic Acids Res 2005 33 17 e141 10.1093/nar/gni139 PMC1236725 16186128

[B15] Sussel L Kalamaras J Hartigan-O'Connor DJ Meneses JJ Pedersen RA Rubenstein JL German MS Mice lacking the homeodomain transcription factor Nkx2.2 have diabetes due to arrested differentiation of pancreatic beta cells Development 1998 125 12 2213 2221 10.1242/dev.125.12.2213 9584121

[B16] Watada H Mirmira RG Kalamaras J German MS Intramolecular control of transcriptional activity by the NK2-specific domain in NK-2 homeodomain proteins Proc Natl Acad Sci USA 2000 97 17 9443 9448 10.1073/pnas.97.17.9443 PMC16883 10944215

[B17] Cissell MA Zhao L Sussel L Henderson E Stein R Transcription factor occupancy of the insulin gene in vivo. Evidence for direct regulation by Nkx2.2 J Biol Chem 2003 278 2 751 756 10.1074/jbc.M205905200 12426319

[B18] Raum JC Gerrish K Artner I Henderson E Guo M Sussel L Schisler JC Newgard CB Stein R FoxA2, Nkx2.2, and PDX-1 regulate islet beta-cell-specific mafA expression through conserved sequences located between base pairs -8118 and -7750 upstream from the transcription start site Mol Cell Biol 2006 26 15 5735 5743 10.1128/MCB.00249-06 PMC1592775 16847327

[B19] Berger MF Philippakis AA Qureshi AM He FS Estep PW Bulyk ML Compact, universal DNA microarrays to comprehensively determine transcription-factor binding site specificities Nat Biotechnol 2006 24 11 1429 1435 10.1038/nbt1246 PMC4419707 16998473

[B20] Anderson KR Torres CA Solomon K Becker TC Newgard CB Wright CV Hagman J Sussel L Cooperative transcriptional regulation of the essential pancreatic islet gene NeuroD1 (beta2) by Nkx2.2 and neurogenin 3 J Biol Chem 2009 284 45 31236 31248 10.1074/jbc.M109.048694 PMC2781522 19759004

[B21] Chen X Hughes TR Morris Q RankMotif++: a motif-search algorithm that accounts for relative ranks of K-mers in binding transcription factors Bioinformatics 2007 23 13 i72 79 10.1093/bioinformatics/btm224 17646348

[B22] Grove CA De Masi F Barrasa MI Newburger DE Alkema MJ Bulyk ML Walhout AJ A multiparameter network reveals extensive divergence between C. elegans bHLH transcription factors Cell 2009 138 2 314 327 10.1016/j.cell.2009.04.058 PMC2774807 19632181

[B23] Doyle MJ Loomis ZL Sussel L Nkx2.2-repressor activity is sufficient to specify alpha-cells and a small number of beta-cells in the pancreatic islet Development 2007 134 3 515 523 10.1242/dev.02763 PMC2805074 17202186

[B24] Prado CL Pugh-Bernard AE Elghazi L Sosa-Pineda B Sussel L Ghrelin cells replace insulin-producing beta cells in two mouse models of pancreas development Proc Natl Acad Sci USA 2004 101 9 2924 2929 10.1073/pnas.0308604100 PMC365721 14970313

[B25] Anderson KR White P Kaestner KH Sussel L Identification of known and novel pancreas genes expressed downstream of Nkx2.2 during development BMC Dev Biol 2009 9 65 10.1186/1471-213X-9-65 PMC2799404 20003319

[B26] Gerrish K Van Velkinburgh JC Stein R Conserved transcriptional regulatory domains of the pdx-1 gene Mol Endocrinol 2004 18 3 533 548 10.1210/me.2003-0371 14701942

[B27] Brodbelt JS Evaluation of DNA/Ligand interactions by electrospray ionization mass spectrometry Annu Rev Anal Chem (Palo Alto Calif) 2010 3 67 87 10.1146/annurev.anchem.111808.073627 20636034

[B28] Wang LH Chmelik R Nirenberg M Sequence-specific DNA binding by the vnd/NK-2 homeodomain of Drosophila Proc Natl Acad Sci USA 2002 99 20 12721 12726 10.1073/pnas.202461199 PMC130527 12232052

[B29] Jiang G Sladek FM The DNA binding domain of hepatocyte nuclear factor 4 mediates cooperative, specific binding to DNA and heterodimerization with the retinoid X receptor alpha J Biol Chem 1997 272 2 1218 1225 10.1074/jbc.272.2.1218 8995424

[B30] Kel AE Niehof M Matys V Zemlin R Borlak J Genome wide prediction of HNF4alpha functional binding sites by the use of local and global sequence context Genome Biol 2008 9 2 R36 10.1186/gb-2008-9-2-r36 PMC2374721 18291023

[B31] Johnson DS Mortazavi A Myers RM Wold B Genome-wide mapping of in vivo protein-DNA interactions Science 2007 316 5830 1497 1502 10.1126/science.1141319 17540862

[B32] Djordjevic M SELEX experiments: new prospects, applications and data analysis in inferring regulatory pathways Biomol Eng 2007 24 2 179 189 10.1016/j.bioeng.2007.03.001 17428731

[B33] Das MK Dai HK A survey of DNA motif finding algorithms BMC Bioinformatics 2007 8 Suppl 7 S21 10.1186/1471-2105-8-S7-S21 PMC2099490 18047721

[B34] Hill JT Chao CS Anderson KR Kaufman F Johnson CW Sussel L Nkx2.2 activates the ghrelin promoter in pancreatic islet cells Mol Endocrinol 2010 24 2 381 390 10.1210/me.2009-0360 PMC2817598 19965928

[B35] Doyle MJ Sussel L Nkx2.2 regulates beta-cell function in the mature islet Diabetes 2007 56 8 1999 2007 10.2337/db06-1766 PMC2805073 17456846

[B36] Steadman DJ Giuffrida D Gelmann EP DNA-binding sequence of the human prostate-specific homeodomain protein NKX3.1 Nucleic Acids Res 2000 28 12 2389 2395 10.1093/nar/28.12.2389 PMC102730 10871372

[B37] Noyes MB Christensen RG Wakabayashi A Stormo GD Brodsky MH Wolfe SA Analysis of homeodomain specificities allows the family-wide prediction of preferred recognition sites Cell 2008 133 7 1277 1289 10.1016/j.cell.2008.05.023 PMC2478728 18585360

[B38] Newburger DE Bulyk ML UniPROBE: an online database of protein binding microarray data on protein-DNA interactions Nucleic Acids Res 2009 37 Database D77 82 10.1093/nar/gkn660 PMC2686578 18842628

