# Supplementary material for: Novel computational analysis of protein binding array data identifies direct targets of Nkx2.2 in the pancreas
Source: BMC Bioinformatics. 2011 Feb 25;12:62. doi: 10.1186/1471-2105-12-62 (PMC3050729; doi:10.1186/1471-2105-12-62)
Supplement: Additional file 1 — Analysis of Dual Core Sites. Octamers containing two adjacent 4-bp core sequences were divided into groups based on content (2 identical cores or a mixture of AAGT and GAGT cores) and relative orientation (inline or reverse complement). Corresponding E-scores form PBM analysis are also shown. Octamers with two reverse complement cores consistently have higher E-scores than inline octamers regardless of content. [file 1471-2105-12-62-S1.PDF]

Additional File 1

	**Same Core**	**Different Cores**
**Inline Orientation**	AAGTAAGT e-score=.23273<br>GAGTGAGT e-score=.35554	AAGTGAGT e-score=.36902<br>GAGTAAGT e-score=.20222
**Reverse Complement Orientation**	AAGTACTT e-score=.49693<br>ACTTAAGT e-score=.47635<br>GAGTACTC e-score=.48698<br>ACTCGAGT e-score=.46437	AAGTACTC e-score=.48686<br>ACTCAAGT e-score=.47887
