# Supplementary figures and images for: Novel computational analysis of protein binding array data identifies direct targets of Nkx2.2 in the pancreas
Source: BMC Bioinformatics. 2011 Feb 25;12:62. doi: 10.1186/1471-2105-12-62 (PMC3050729; doi:10.1186/1471-2105-12-62)

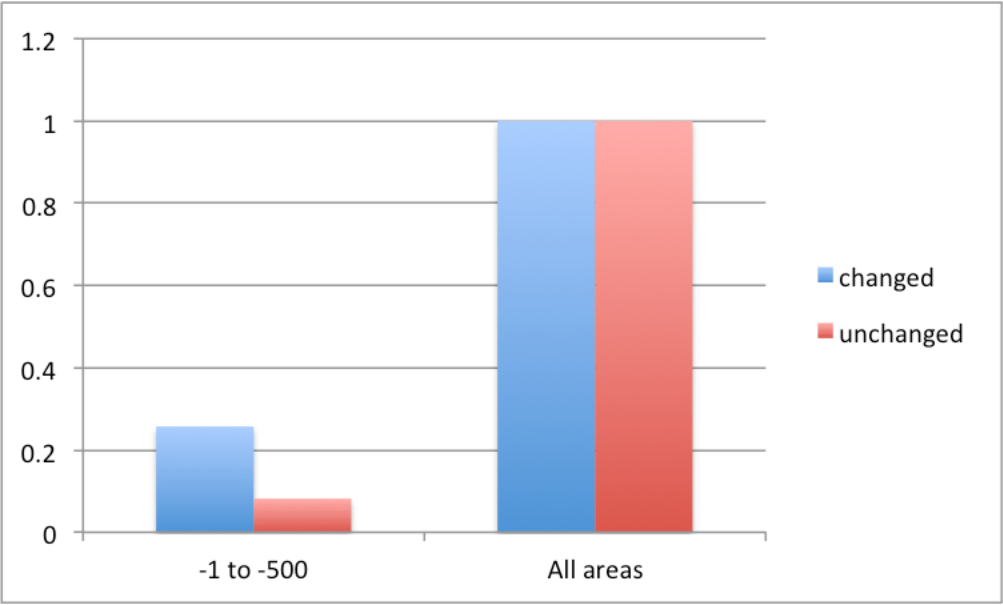

Supplement: Additional file 2 — Enrichment of sites in proximal promoter regions of genes differentially expressed in the Nkx2.2 null embryo. Putative Nkx2.2 binding sites were predicted (PBM-mapping score > 0.40) in promoter regions of 100 randomly chosen genes from genes with no differential expression between the Nkx2.2 null and wildtype embryos and compared to the 35 differentially expressed genes. The distance from the transcriptional start site of the closest predicted site was then calculated. Differentially expressed genes were more likely to have sites within 500 bp of the transcriptional start site (P = 0.02). No statistically significant difference was seen in the other regions. [file 1471-2105-12-62-S2.PDF]

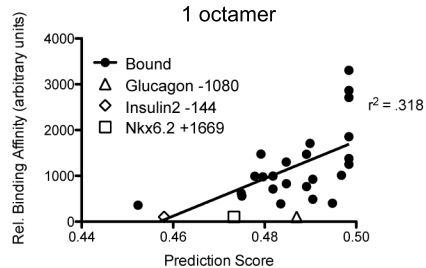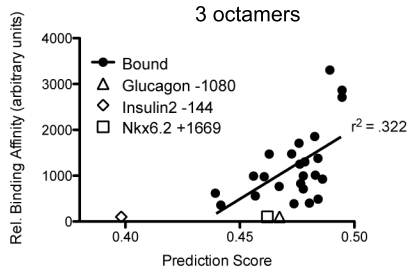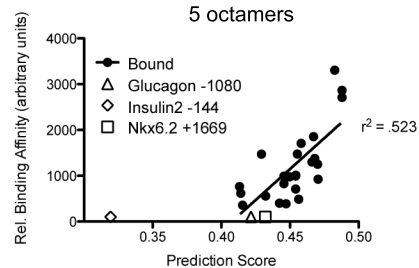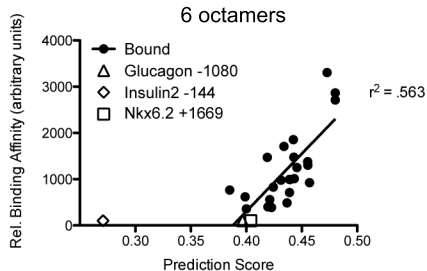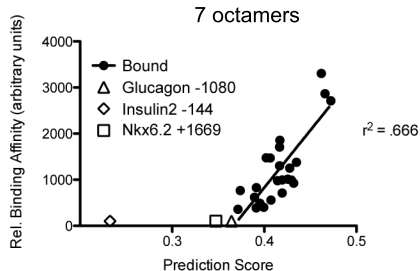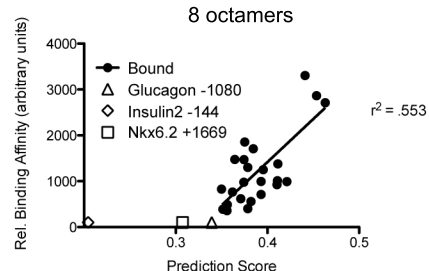

Supplement: Additional file 3 — Optimization of moving average of E-score values. A moving average of E-scores containing 1, 3, 5, 6, 7, or 8 overlapping octomers was calculated and compared to relative binding affinity of each site. R-squared values are plotted next to each plot. The three sites that did not bind in our EMSA analysis are plotted along the x-axis to show their predicted scores compared to bound sites, but were not used to calculate r-squared values. [file 1471-2105-12-62-S3.PDF]

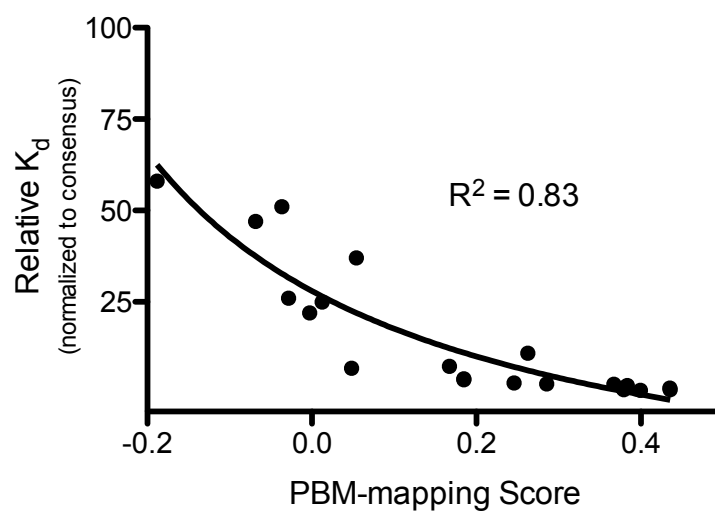

Supplement: Additional file 4 — PBM-mapping scores are highly correlated with Kd values for the Nkx2.2 drosophila homolog vnd. Previously published Kd values for 22 vnd binding sites were plotted against their respective PBM-mapping scores. Non-linear regression was performed using a previously derived equation for the expected relationship between PBM-mapping scores and Kd values (see Methods). [file 1471-2105-12-62-S4.PDF]

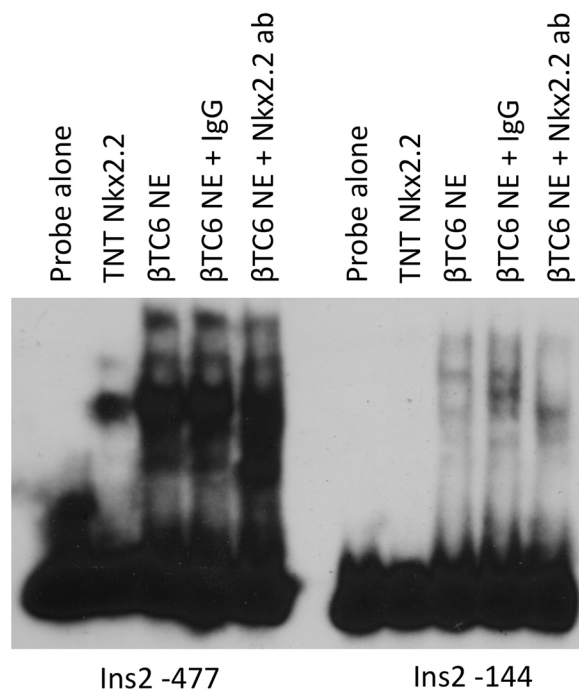

Supplement: Additional file 5 — An Nkx2.2 containing complex forms on the Ins2 -144 site. Longer exposure (48 hrs) of the EMSA analysis of putative Nkx2.2 binding sites in the Ins2 promoter shown in Figure 6. Probes were incubated with in vitro translated Nkx2.2 or βTC6 nuclear extract. Supershifts were done using the monoclonal Nkx2.2 antibody. [file 1471-2105-12-62-S5.PDF]
