# Supplementary material for: Novel computational analysis of protein binding array data identifies direct targets of Nkx2.2 in the pancreas
Source: BMC Bioinformatics. 2011 Feb 25;12:62. doi: 10.1186/1471-2105-12-62 (PMC3050729; doi:10.1186/1471-2105-12-62)
Supplement: Additional file 6 — Confirmation of previously tested Hnf4α sites. PBM-mapping scores were generated for 18 positive and 12 negative Hnf4α sites that were previously published (28). At a threshold of 0.26, 16 of the 18 confirmed sites were predicted while all of the negative sites were not predicted. The two sites that were not predicted, but were bound in EMSA analysis, are highlighted in Bold. [file 1471-2105-12-62-S6.PDF]

Gene	Position[1]	Sequence	Bound (EMSA)[2]	PBM-mapping score
179TCF1/HNF1	-265	AAGGCTGAAGTCCAAAGTTCAGTCCCTTC	Y	0.414
APOB	-86	GGAAAGGTCCAAAGGGCGCCTTG	Y	0.429
SERPINA1/AAT	-134	CAACAGGGGCTAAGTCCACTGGC	Y	0.339
AGT	-429	TGCAGAGGGCAGAGGGCAGGGGA	Y	0.278
APOC3	-93	GGCGCTGGGCAAAGGTCACCTGC	Y	0.419
CYP2D6	-69	AGCAGAGGGCAAAGGCCATCATC	Y	0.338
TF	-76	ACGGGAGGTCAAAGATTGCGCCC	Y	0.362
ALDH2	-332	CATTGGGGTCAAAGGCACACATT	Y	0.408
APOC2	-159	TGTCTAGGCCAAAGTCCTGGCCA	Y	0.261
PCK1	-455	GGTCACAGTCAAAGTTCATGGGA	Y	0.354
NCOA2	-485	ATGGGAGGGCAAAGGGCAATGCC	Y	0.388
TFF2	-495	AAGATGGGACAAAGGGCATCGTG	Y	0.328
**CHEK1**	**5**	**AGTGGTGGGCAAAGGACAGTCCG**	**Y**	**0.209**
**CD63**	**-182**	**CTGCAGGAGCAAAGGACAGAAGT**	**Y**	**0.195**
SH3GL2	-393	CGCCAGGCTCAAAGGGCAGGAGG	Y	0.326
RND2		AGGGCAGGTCAGAGTTCAAGCGA	Y	0.373
ESRRBL1	63	CAGAACGGACAGAGTCCAGCGTG	Y	0.308
DDB1	-295	GGGGAAGGGCAAAGGGCGCGGAA	Y	0.377
NEUROG3	-225	GATTCCGGACAAAGGGCCGGGGT	N	0.238
IL6	-149	ACTAGGGGGAAAAGTGCAGCTTA	N	0.145
AZI2	-217	GGACCCCCCAAAAGGACACTGAG	N	0.209
CFL2	-676	CGAGGCGAGAAAAGCCCCCCGCA	N	0.002
GPHN	733	GACTGAGAGGAAAGGATAGCACA	N	-0.010
C14orf119	-610	CAAGCGGCTCAAAGGGGTGAGGA	N	0.242
PPP1R3C	-142	CGAGACGTGCAGAGAGCTATCTG	N	-0.058
AKR1C3	-481	GAAAATGTAAAAAGGCAAATATT	N	0.095
NPAS2	-395	GAGCCGGCCCAGAGGAGAGGCAA	N	-0.050
SAG	-106	CCTGGGAGACAGAGCAAGACTCC	N	-0.024
CLCN3	-377	AGCGTCACGCAGAGTTCGGATCC	N	0.201
CBX3	-525	GCGGAAGGCTAGAGTCCTGCTAG	N	0.120

1. Position relative to transcriptional start site.
2. See Kel et al. 2008 (28)
