# Supplementary material for: Novel computational analysis of protein binding array data identifies direct targets of Nkx2.2 in the pancreas
Source: BMC Bioinformatics. 2011 Feb 25;12:62. doi: 10.1186/1471-2105-12-62 (PMC3050729; doi:10.1186/1471-2105-12-62)
Supplement: Additional file 7 — List of probes used in EMSA analysis. Forward and reverse single stranded oligos that were annealed to form double stranded DNA probes with 5' overhangs. Probes were then labeled by Klenow extension to insert a 32P containing dCTP (see Methods). [file 1471-2105-12-62-S7.PDF]

Probe	Sequence
Chgb -1529 Forward	GAACAAACAC AGGGTGACTC ATTGAAGTGT GATGCATGGC TAAAAGCAGA
Chgb -1529 Reverse	AGTTCTGCTT TTAGCCATGC ATCACACTTC AATGAGTCAC CCTGTGTTTG
Chgb -217 Forward	TGAGGTTAAA AGAGAGAGAG AATTTTGAAG TGTATCCTTT GGC
Chgb -217 Reverse	AGGCCAAAGG ATACACTTCA AAATTCTCTC TCTCTTTTAA CC
Frzb -2290 Forward	AGTCCAAATA TCTTAAGGAG ATAAACCACT TGAGAGGAGA CTTAATTC
Frzb -2290 Reverse	TTGAGAATTA AGTCTCCTCT CAAGTGGTTT ATCTCCTTAA GATATTTGG
Gcg -1080 Forward	AGACCATTGA AACAACTGGA GGAGTACTCT GACTGAACTT AATTCTTCAT
Gcg -1080 Reverse	AGAATGAAGA ATTAAGTTCA GTCAGAGTAC TCCTCCAGTT GTTTCAATGG
Gcg -280 Forward	ACGAAAAACT GCTAAAGTTC TCTCAAGTGA ATTTTGACGT CAAATGAGCC TAG
Gcg -280 Reverse	AGACTAGGCT CATTTGACGT CAAAATTCAC TTGAGAGAAC TTTAGCAGTT TTT
Gcg -432 Forward	AGTACACACA TATCAATAAC CCACTCATCC ACATTGTATG GAATAAATTT GTAT
Gcg -432 Reverse	AGAATACAAA TTTATTCCAT ACAATGTGGA TGAGTGGGTT ATTGATATGT GTGT
Iapp -1184 Forward	AGTGTAAAAA ATAAATTAAT TTTAAAAAAA ACACTTAAAC GTGAACACAT
Iapp -1184 Reverse	TGTATGTGTT CACGTTTAAG TGTTTTTTTT AAAATTAATT TATTTTTTAC
Iapp -1355 Forward	TGTCCTCAGG CCGCTACATA AAGGCACTCA AGAGACTGGA GGCCCCAGGG AGTTTGGAGG
Iapp -1355 reverse	TGACCTCCAA ACTCCCTGGG GCCTCCAGTC TCTTGAGTGC CTTTATGTAG CGGCCTGAGG
Iapp -1955 Forward	GTTAAGCTGG TATGGCTAGT TAAGTGGTTA TAGCTGACAT ATAATGTCT
Iapp -1955 Reverse	TGAAGACATT ATATGTCAGC TATAACCACT TAACTAGCCA TACCAGCTT
Iapp +479 Forward	TGTCCTCCTC ATCCTCTCTG TGGCACTGAA CCACTTGAGA GCTACACCTG
Iapp +479 Reverse	TGACAGGTGT AGCTCTCAAG TGGTTCAGTG CCACAGAGAG GATGAGGAGG
Ins -144 Forward	TGCTTTCTGC AGACCTAGCA CCAGGCAAGT GTTTGGAAAC TGCAGCT
Ins -144 reverse	CTGAAGCTGC AGTTTCCAAA CACTTGCCTG GTGCTAGGTC TGCAGAA
Ins -471 forward	AAGCAGAACT CAGGCAGCAA GGTACTTAAT GGTCCCTCCT TCTCCATC
Ins -471 Reverse	AGAGATGGAG AAGGAGGGAC CATTAAGTAC CTTGCTGCCT GAGTTCT
Irs4 -111 Forward	CCGCCTAGGC CCGCGTCCCC GCCCACTTCA CTGGGCTCAA GGCAGTGG
Irs4 -111 reverse	TGCCCACTGC CTTGAGCCCA GTGAAGTGGG CGGGGACGCG GGCCTAGG
Irs4 +1495 Forward	AGCCCTGGCT ACTGGAACCT TGGCCACTTG AGCCCCGTCC ACCTCCTGAG CCC
Irs4 +1495 reverse	CCGGGGCTCA GGAGGTGGAC GGGGCTCAAG TGGCCAAGGT TCCAGTAGCC AGG
Mafa Forward	TGTAACCAGG AGGCAGCCCC TCCAGCAAGC ACTTCAGTGT GCTCAGTGGG
Mafa reverse	AACAGCCCCA CTGAGCACAC TGAAGTGCTT GCTGGAGGGG CTGCCTCCTG G
Ngn3 -506 Forward	CGCTCCTCCC AGCTGCCAGC CAAGAAGACA CTTGACTCCT TGATCGCTGG T
Ngn3 -506 Reverse	TGAACCAGCG ATCAAGGAGT CAAGTGTCTT CTTGGCTGGC AGCTGGGAGG A
Nkx2.2 -1502 Forward	GCTGCAAGTT TGCTACATAC CACTTGTTCG CCCCACTTAA CATCAGGAGT GGGCTT
Nkx2.2 -1502 Reverse	GCTAAGCCCA CTCCTGATGT TAAGTGGGGC GAACAAGTGG TATGTAGCAA ACTTGC
Nkx2.2 -188 Forward	CGCGTCGCTC TCGAGTCCAC ACACTTGAAA AGAGCCGTTT TAACAAATT
Nkx2.2 -188 Reverse	ATGCAATTTG TTAAAACGGC TCTTTTCAAG TGTGTGGACT CGAGAGCGAC
Nkx2.2 -377 forward	ACGTGTGGGC GGGTCTTGGG AGTCAAGTGG ATGAAGACAG TATTTG
Nkx2.2 -377 Reverse	CTGCAAATAC TGTCTTCATC CACTTGACTC CCAAGACCCG CCCAC
Nkx2.2 -716 Forward	GTCAATATTT TGGTTGAAGC TTAAGGATGA GTACTAGAAA TGACAAG
Nkx2.2 -716 Reverse	TGACTTGTCA TTTCTAGTAC TCATCCTTAA GCTTCAACCA AAATATT
Nkx2.2 AAGT Forward	TCGACGCGAT TTTTCAAGTG GTTTTCAGTA GC
Nkx2.2 AAGT Reverse	AGCTGCTACT GAAAACCACT TGAAAAATCG CG
Nkx2.2 GAGT Forward	TCGACGCGAT TTTTCGAGTG GTTTTCAGTA GC
Nkx2.2 GAGT Reverse	AGCTGCTACT GAAAACCACT CGAAAAATCG CG
Nkx2.2 No Core Forward	TCGACGCGAT TTTTCGGTTT TCAGTAGC

Nkx2.2 No Core Reverse	AGCTGCTACT GAAAACCGAA AAATCGCG
Nkx6.2 -1441 Forward	AGCCACTTTA TGGCGGGAAC TGGAAATAAG TGCTGTGGTC CCGCTGACTT CT
Nkx6.2 -1441 Reverse	TGCAGAAGTC AGCGGGACCA CAGCACTTAT TTCCAGTTCC CGCCATAAAG TG
Nkx6.2 +669 forward	CCGAATCCCG CGCGGGCCAC TTACCGGAGC CGGCCAGTCG CGGGTCCCTC
Nkx6.2 +669 reverse	CTGGAGGGAC CCGCGACTGG CCGGCTCCGG TAAGTGGCCC GCGCGGGATT
pdx1 -5877 site for	TGCTCATGTG GGCAGAATTA AGTGGAATTA GCTAACAAAT TATATAAAAT
Pdx1 -5877 site rev	TGAATTTTAT ATAATTTGTT AGCTAATTCC ACTTAATTCT GCCCACATGA
Spock3 -1041 Reverse	GCAACAGGTG TGTCCCGTAT TCTGAGTACT TTGTTCTCAC TCGGGTCATA
Spock3 -1044 Forward	AGTTATGACC CGAGTGAGAA CAAAGTACTC AGAATACGGG ACACACCTGT
Tm4sf4 -1723 forward	GCCATTAGTG CCAATGACCC AGCACTCGAG GGTAGGGGGA GCACAGC
Tm4sf4 -1723 reverse	ACTGGCTGTG CTCCCCCTAC CCTCGAGTGC TGGGTCATTG GCACTAATG
Tm4sf4 -5 Forward	CTGAAGGCCT GCCGTAGTTG AGAAGTGAAG TGTCTCCAAG GTTCAAAGAA CT
Tm4sf4 -5 Reverse	CAGAGTTCTT TGAACCTTGG AGACACTTCA CTTCTCAACT ACGGCAGGCC TT
Tm4sf4 +555 Forward	AGCCCAGAGA ACCAAGCTAA TAGCCACTTG ATTATTTTAC TCTAGTCAAA TTGTG
Tm4sf4 +555 Reverse	TGCCACAATT TGACTAGAGT AAAATAATCA AGTGGCTATT AGCTTGGTTC TCTGG
Tm4sf4 +912 Forward	CGGCTGTTAG GTCTTGCCTG CCCCACTTAA GCCCCTGAGA CCTGAGGTCT
Tm4sf4 +912 Reverse	TGAAGACCTC AGGTCTCAGG GGCTTAAGTG GGGCAGGCAA GACCTAACAG C
