# Supplementary material for: Novel computational analysis of protein binding array data identifies direct targets of Nkx2.2 in the pancreas
Source: BMC Bioinformatics. 2011 Feb 25;12:62. doi: 10.1186/1471-2105-12-62 (PMC3050729; doi:10.1186/1471-2105-12-62)
Supplement: Additional file 8 — List of primers used for qPCR reactions. PCR primers were designed to amplify an approximately 200 bp region flanking predicted Nkx2.2 binding sites (see Methods). [file 1471-2105-12-62-S8.PDF]

**Primer**	**Sequence**
Chgb -217 For	CACCAATTATGTGTGCTCCAA
Chgb -217 Rev	GGAATCTCCTACCCGACGTA
Chgb -1529 For	GGGAACAAACACAGGGTGAC
Chgb -1529 Rev	TCACTACCCTATTCCCATTTTCA
Frzb -2290 For	TCCGAATTTTGGGTTTGTTG
Frzb -2290 Rev	AAAACTGGCTGGTGGAAATG
Gcg -280/-432 For	TCTCCCCACAAAGAGAATACAAA
Gcg -280/-432 Rev	CCCTTGATTTGGTATTTGGC
Gcg -1080 For	GTAGCTCCACACCCACCAGT
Gcg -1080 Rev	TGACAAGACCACAGCGTTTC
Iapp -1955 For	CCAGTGGTTAAGCTGGTATGG
Iapp -1955 Rev	TATTGCAAATGCCACTCCTG
Iapp -1184/-1355 For	GAGAAGCTGAAAATCGACGC
Iapp -1184/-1355 Rev	GGCCTCCAGTCTCTTGAGTG
Iapp +479 For	CAGCTGTCCTCCTCATCCTC
Iapp +479 Rev	TCTCATAGCCAGGATTTGCTT
Irs4 -111 For	GACGGTCACGTGTTGTTTTG
Irs4 -111 Rev	GATGCACCGTGGTTTTAAGG
Ngn3 -506 For	GGTTGCACACACATTTCCTG
Ngn3 -506 Rev	TCTTTTGGCTCAGAGAGGGA
Nkx2-2 -188/-377 For	CGGCTCTTTTCAAGTGTGTG
Nkx2-2 -188/-377 Rev	GTGAAATTGTGGGTTTTGGG
Nkx2-2 -716 For	CTGGCATGTCCAAGCCTATT
Nkx2-2 -716 Rev	GCTGGTGGTTCCCTAAACAA
Nkx2-2 -1502/-1516 For	GGACTAAGGCAACCCAAACA
Nkx2-2 -1502/-1516 Rev	GAGGTACGAGGCTGCAAGTT
Pdx1 -5877 For	CAAGCACACAGTAGGTGTTCTC
Pdx1 -5877 Rev	TGCCTCTGACTGTGTCCCACT
Spock3 -1044 For	ATCATCTAAAAGTTATGACCCGAG
Spock3 -1044 Rev	TGAATTACATATGTCAGGCAAGC
Tm4sf4 -1723 For	GGGAGATGATGCAGTGGGTACG
Tm4sf4 -1723 Rev	TTCAGGGGCAGTCACACTTAGAC
Tm4sf4 -5 For	GGCCTGCCGTACTTGAGAAG
Tm4sf4 -5 Rev	CACAGGAAAGCACAGAGATCAAAGG
Tm4sf4 +483/+555 For	CCCTTTCTATTCGCGGCTGG
Tm4sf4 +483/+555 Rev	CTTACAGCTTCTGTGTCCCTTCAT
Mafa For	CACCCCAGCGAGGGCTGATTTAATT
Mafa Rev	AGCAAGCACTTCAGTGTGCTCAGTG
GapdH For	CGCATCTTCTTGTGCAGTGCCAG
GapdH Rev	TACGGGACGAGGCTGCAGGAG
